# Examining the Antioxidant and Superoxide Radical Scavenging Activity of Anise, (*Pimpinella anisum* L. Seeds), Esculetin, and 4-Methyl-Esculetin Using X-ray Diffraction, Hydrodynamic Voltammetry and DFT Methods

**DOI:** 10.3390/ph17010067

**Published:** 2023-12-31

**Authors:** Miriam Rossi, Francesco Caruso, Natalie Thieke, Stuart Belli, Alana Kim, Elisabetta Damiani, Camilla Morresi, Tiziana Bacchetti

**Affiliations:** 1 Department of Chemistry, Vassar College, Poughkeepsie, NY 12604, USA; caruso@vassar.edu (F.C.); nthieke@vassar.edu (N.T.);; 2 Department of Life and Environmental Sciences, Polytechnic University of Marche, Via Brecce Bianche, 60131 Ancona, Italy; e.damiani@univpm.it (E.D.); t.bacchetti@staff.univpm.it (T.B.)

**Keywords:** coumarin, voltammetry, X-ray diffraction, π–π interaction, DFT, superoxide

## Abstract

*Pimpinella anisum* L., or anise, is a plant that, besides its nutritional value, has been used in traditional medical practices and described in many cultures in the Mediterranean region. A possible reason for anise’s therapeutic value is that it contains coumarins, which are known to have many biomedical and antioxidant properties. HPLC analysis in our laboratory of the anise extract shows the presence of the coumarin esculetin. We used a hydrodynamic voltammetry rotating ring–disk electrode (RRDE) method to measure the superoxide scavenging abilities of anise seeds and esculetin, which has marked scavenging activity. A related coumarin, 4-methyl-esculetin, also showed strong antioxidant activity as measured by RRDE. Moreover, this study includes the X-ray crystal structure of esculetin and 4-methyl-esculetin, which reveal the H-bond and the stacking intermolecular interactions of the two coumarins. Coordinates of esculetin crystal structure were used to perform a DFT study to arrive at the mechanism of superoxide scavenging. Besides performing a H(hydroxyl) abstraction in esculetin position 6 by superoxide, the scavenging also includes the presence of a second superoxide radical in a π–π approach. Both rings of esculetin were explored for this attack, but only the pyrone ring was effective. As a result, one product of esculetin scavenging is H_2_O_2_ formation, while the second superoxide remains π–π trapped within the pyrone ring to form an esculetin-η-O_2_ complex. Comparison with other coumarins shows that subtle structural differences in the coumarin framework can imply marked differences in scavenging. For instance, when the catechol moiety of esculetin (position 6,7) is shifted to position 7,8 in 4-methyl-7,8-dihydroxy coumarin, that coumarin shows a superoxide dismutase action, which, beside H_2_O_2_ formation, includes the formation and elimination of a molecule of O_2_. This is in contrast with the products formed through esculetin superoxide scavenging, where a second added superoxide remains trapped, and forms an esculetin-η-O_2_ complex.

## 1. Introduction

*Pimpinella anisum* L. (*P. anisum*), commonly known as anise, is an annual herb belonging to the Umbelliferae family (Apiaceae). It is one of the oldest plant species native to Mediterranean countries. *P. anisum* fruits (anise seeds) have been used widely as both a food and as an ingredient in ethnomedicinal remedies in many parts of the world [1]. More recently, the health benefits of anise have been reviewed [2,3]. The Apiaceae family, besides anise, includes widely used foods in cooking preparations around the world such as carrots, celery, coriander, cumin, fennel, and parsley. Also, many traditional liqueurs are produced using *P. anisum*, such as sambuca, anisette, ouzo, and pastis. Per se, the presence of some of these foods in the human diet is almost guaranteed. Anise has a characteristic aromatic odor and taste similar to licorice, star anise, and fennel that is easily recognizable and that has been identified with the compound anethole [4]. Members of the Apiaceae family also contain other interesting compounds having bioactive properties, including coumarins. In fact, the fruits of the Apiaceae family of plants are, by far, the major source of coumarins [5,6]. Coumarins are a family of secondary metabolites in higher plants exhibiting vast structural diversity. These secondary metabolites carry out numerous functions and are produced in response to environmental stresses as well as being implicated in plant defense mechanisms against insects, herbivores, and microorganisms [7]. In humans, coumarins are known to have extensive therapeutic properties [6,8,9].

Earlier studies on diverse coumarins carried out in our laboratory [10,11] have highlighted their antioxidant activity. Esculetin has demonstrated antibacterial, anti-inflammatory, antioxidant, and antitumor activities [12]. A derivative, 4-methyl-esculetin, has potent anti-inflammatory activity [13], and its antioxidant activity was previously studied [14,15,16]. Our laboratory carried out computational DFT studies on a related 4-methyl coumarin, 4-methyl-7,8-dihydroxy coumarin (4-methyldaphnetin) [11].

The aim of our work was to investigate the antioxidant capacity of anise extract, esculetin, and 4-methyl-esculetin. Antioxidant activity in our laboratory was measured using electrochemical techniques to determine the superoxide scavenging capacity of the antioxidants. The superoxide anion in the body is mostly produced as a consequence of cellular respiration, an essential process by which complex molecules are broken down to smaller useful ones while releasing ATP. It is also an important component of the immune defense system. Therefore, the concentration of the superoxide radical is crucial to both health and disease states. Normally, the body regulates this concentration by using redox metalloenzymes, the superoxide dismutase (SOD) enzymes [17,18]. However, in events of biological stress, the SOD enzyme action needs to be enhanced by the presence of molecular collaborators, many of which are polyphenolic compounds found in edible plants. To understand the relationship between molecular structure and the chemical activity of the two coumarin molecules, we crystallized esculetin and 4-methyl-esculetin and used single-crystal X-ray diffraction to obtain information on the intermolecular interactions. Last, by performing computational studies using DFT methods, we describe a chemical mechanism by which these two coumarin molecules can act as efficient superoxide scavengers.

## 2. Materials and Methods

Esculetin (6,7-dihydroxycoumarin), C_9_H_6_O_4_, and 4-methyl-esculetin (6,7-dihydroxy-4-methylcoumarin), C_10_H_8_O_4_, were obtained from Indofine Chemical Co. (Hillsborough, NJ 08844, USA); tetrabutylammonium bromide, TBAB, and dimethyl sulfoxide, DMSO, anhydrous, ≥99.9% from Sigma-Aldrich (St. Louis, MO, USA).

### 2.1. Plant Material and Extraction

The seeds of *Pimpinella anisum* L., were kindly supplied by the farm Carboni, Castignano (Ascoli Piceno), Marche, Italy. The seeds were finely chopped in a food processor. For the extract, 25 g of anise powder was suspended in 500 mL of boiling water by magnetic stirrer for 15 min. Then, the extract was filtered over Whatman No. 1 paper and stored in aliquots at −20 °C [19].

### 2.2. HPLC Study

Analysis of the anise sample was carried out on a HP Agilent HPLC system consisting of a Model Agilent 1100 series with a Model Agilent series G-1315A DAD detector. Separations were carried out with an Agilent Eclipse XDB-C18 column (250 4.6 mm i.d., 5 um). Isocratic elution was performed with a mixture of methanol: 0.1% formic acid in water (30:70 *v*/*v*) at a flow rate of 1.0 mL min^−1^. The sample injection volume for both was 20 µL. Analyses were carried out at an ambient temperature (20 °C), monitoring at 254 nm and 350 nm.

### 2.3. Single Crystal X-ray Diffraction Analysis

X-ray crystallographic analyses on suitable crystals of esculetin and 4-methyl-esculetin were carried out using a Bruker APEX-II CCD diffractometer. The X-ray intensity data were measured using MoKα radiation (λ = 0.71073 Å) at 125 K. Crystal data, data collection, and structure refinement details for the two compounds are summarized in Table 1.

#### 2.3.1. Esculetin

Esculetin was recrystallized after 4 days from a 1:1 ethanol/H_2_O solution. A clear colorless long prism crystal of C_9_H_6_O_4_, of approximate dimensions 0.150 mm × 0.190 mm × 0.340 mm, was used for X-ray crystallographic analysis. A total of 2550 frames were collected. The total exposure time was 14.17 h. The frames were integrated with the Bruker SAINT [20] software package, using a narrow-frame algorithm. The integration of the data using a monoclinic unit cell yielded a total of 17,336 reflections to a maximum θ angle of 30.50° (0.70 Å resolution), of which 2201 were independent (average redundancy 7.876, completeness = 100.0%, R_int_ = 2.38%, R_sig_ = 1.13%) and 2027 (92.09%) were greater than 2σ(F^2^). The final cell constants in Table 1 are based upon the refinement of the XYZ-centroids of 9885 reflections above 20 σ(I) with 5.066° < 2θ < 63.18°. Data were corrected for absorption effects using the multi-scan method (SADABS) [20]. The ratio of minimum to maximum apparent transmission was 0.936. The calculated minimum and maximum transmission coefficients (based on crystal size) are 0.9570 and 0.9810.

The structure was solved and refined using the Bruker SHELXTL software package, [21] and Olex2 [22] using the space group *P*2*_1_/c*, with Z = 4 for the formula unit, C_9_H_6_O_4_. The final anisotropic full-matrix least-squares refinement on F^2^ with 142 variables converged at R1 = 3.60% for the observed data and wR2 = 10.9% for all data. The largest peak in the final difference electron density synthesis was 0.538 e^−^/Å^3^ and the largest hole was −0.277 e^−^/Å^3^ with an RMS deviation of 0.062 e^−^/Å^3^.

Crystal structure drawings were made using Mercury, developed at the Cambridge Crystallographic Data Centre, CCDC [23]. All nonhydrogen atoms were refined anisotropically. The positions of all the hydrogen atoms were found in the difference Fourier synthesis electron density map and refined freely.

#### 2.3.2. 4-Methyl-Esculetin

4-Methyl-esculetin was recrystallized from a 1:1 ethanol/H_2_O solution. A colorless plate-like specimen of C_10_H_8_O_4_, approximate dimensions 0.100 mm × 0.170 mm × 0.270 mm, was used for the X-ray crystallographic analysis. The X-ray intensity data were measured (λ = 0.71073 Å). A total of 2550 frames were collected. The total exposure time was 14.17 h. The frames were integrated with the Bruker SAINT software package [20] using a narrow-frame algorithm. The integration of the data using a triclinic unit cell yielded a total of 9707 reflections to a maximum θ angle of 30.03° (0.71 Å resolution), of which 2339 were independent (average redundancy 4.150, completeness = 99.8%, R_int_ = 2.05%, R_sig_ = 1.89%) and 1861 (79.56%) were greater than 2σ(F^2^). The final cell constants reported in Table 1 are based upon the refinement of the XYZ-centroids of 5112 reflections above 20 σ(I) with 4.600° < 2θ < 62.82°. Data were corrected for absorption effects using the multi-scan method (SADABS) [20]. The ratio of minimum to maximum apparent transmission was 0.925. The calculated minimum and maximum transmission coefficients (based on crystal size) are 0.9670 and 0.9880.

The structure was solved and refined using the Bruker SHELXTL software package [21] and Olex2 [22] using the space group *P* − 1, with Z = 2 for the formula unit, C_10_H_8_O_4_. The final anisotropic full-matrix least-squares refinement on F^2^ with 160 variables converged at R1 = 3.97% for the observed data and wR2 = 11.07% for all data. The largest peak in the final difference electron density synthesis was 0.472 e^−^/Å^3^ and the largest hole was −0.280 e^−^/Å^3^ with an RMS deviation of 0.058 e^−^/Å^3^.

Crystal structure drawings were made using Mercury [23]. All nonhydrogen atoms were refined anisotropically. The positions of all the hydrogen atoms were found in the difference Fourier synthesis electron density map and refined freely.

### 2.4. Hydrodynamic Voltammetry (RRDE)

For the experiment using the anise, a sample was dissolved in anhydrous DMSO. For the experiments regarding the two coumarin molecules, esculetin and 4-methyl-esculetin, were used as obtained from Indofine Chemicals. The compounds were dissolved in anhydrous DMSO. Concentration of esculetin and 4-methyl-esculetin were 0.05 M, whereas 0.02 g of anise seeds were dissolved in 10 mL of DMSO. For all the three experiments, the electrochemical cell containing a solution of 0.1 M previously dried TBAB, dissolved in 50 mL DMSO, anhydrous, ≥99.9%, was bubbled with dry O_2_/N_2_ (35%/65%) for five minutes to establish the required dissolved molecular oxygen level.

The TBAB salt used in electrochemical studies is sustainable, low-cost, chemically stable, and easily available. The rotation setting used for the rotation of the Au/Au disk electrode was fixed at 1000 rpm, and the potential sweep was applied to the disk from 0.2 V to −1.2 V and then reversed to 0.2 V, while the potential of the ring electrode was held invariable at 0.0 V. The disk voltage sweep rate was positioned at 25 mV/s. Initially, a blank solution containing all reagents except the antioxidant made up of bubbled O_2_, the electrolyte TBMB and DMSO alone was run, and the “efficiency”—the ratio of the ring current/disk current—was computed. An aliquot sample of DMSO-dissolved anise antioxidant was then introduced to the electrochemical cell, as indicated in Section 3. This sample solution in the voltaic cell was then bubbled with the O_2_/N_2_ gas mixture for 5 min, after which an updated voltammogram was recorded, and the matching efficiency was obtained. In this manner, the rate at which the increasing concentrations of the antioxidant sequester the generated superoxide radicals during the electrochemical reaction was determined as each subsequent sample of antioxidant was added. Aftermath software, release 1.6.10523 (Pine Research Instrumentation, Durham, NC, USA), was used to document the results from each run, represented as voltammograms showing the current vs. potential plots. The results were analyzed using Microsoft Excel. The volume amount used in each of the anise aliquots is indicated in the related RRDE plots, whereas for esculetin and 4-methyl-esculetin, the final concentration is indicated in their related plots. Finally, the decreasing slope of the curve describes how the increasing addition of small amounts of the antioxidant produces the overall decrease in efficiency and serves as a quantitative measure of the antioxidant action of anise sample and the two coumarins. Any reduction in the collection efficiency was projected to be due to the amount of superoxide eliminated by the antioxidant. This method was developed in our laboratory [24]. In our RRDE voltammetry experiment, the generation of the superoxide radical anions occurs through a reduction of the neutral oxygen molecule detected around −0.6 V at the disk electrode, Equation (1), while the reverse oxidation reaction, Equation (2), of the residual superoxide radicals (those that remain unreacted) are detected at the ring electrode.

Reduction of molecular oxygen, electron gain, to form superoxide radical anion, occurring at the disk electrode:

O_2_ + e^−^ → O_2_^•−^ (1)

Reverse of (1): oxidation of superoxide radicals, electron release, to form molecular oxygen, at the ring electrode:

O_2_^•−^ → O_2_ + e^−^ (2)

### 2.5. Computational Study

Calculations were run using BIOVIA Materials Studio DMoL^3^, a modeling program [25] that uses density functional theory (DFT) to simulate chemical processes. We used DMoL^3^ to perform simulated experiments, and calculate energy, geometry, and frequencies, to understand and describe the fundamental chemical reactions involved in scavenging the superoxide radical anion [26]. We employed the double-numerical polarized (DNP) basis set including all the occupied atomic orbitals plus a second set of valence atomic orbitals, as well as polarized d-valence orbitals [27]. Correlation generalized gradient approximation (GGA) was used, including BLYP correlation and Becke exchange [28]. All electrons were treated explicitly, and the real space cutoff of 5 Å was set for the numerical integration of the Hamiltonian matrix elements. The self-consistent field convergence criterion was established for the root mean square variation in the electronic density to be less than 10^−6^ electron/Å^3^. Solvent effects were not included in these DFT calculations. The convergence criteria applied during geometry optimization were 2.72 × 10^−4^ eV for energy and 0.054 eV/Å for force.

## 3. Results and Discussion

The electrochemical technique [24] developed in our laboratory is very useful to study the scavenging of superoxide by small organic molecules found in fruit and vegetables. We have used it to describe the behavior of flavonoids [24,29,30], chalcones [31], coumarins [12], and quinones [32,33,34]. We also investigated the scavenging capacity of several integral natural products along with phytochemicals contained in the same natural products. By using DFT computational techniques, we also obtained the related chemical reaction mechanisms by which scavenging occurs. For instance, we studied olive oil and its component tyrosol [35], propolis and galangin [30], black seed oil and thymoquinone [33]. In keeping with these studies, we analyzed the scavenging of superoxide by anise and one of its components, esculetin. Earlier work on anise extract showed that it had stronger superoxide scavenging activity than butylated hydroxytoluene (BHT) and α-tocopherol but less than butylated hydroxyanisole (BHA) [19].

### 3.1. HPLC

**Figure 1:**
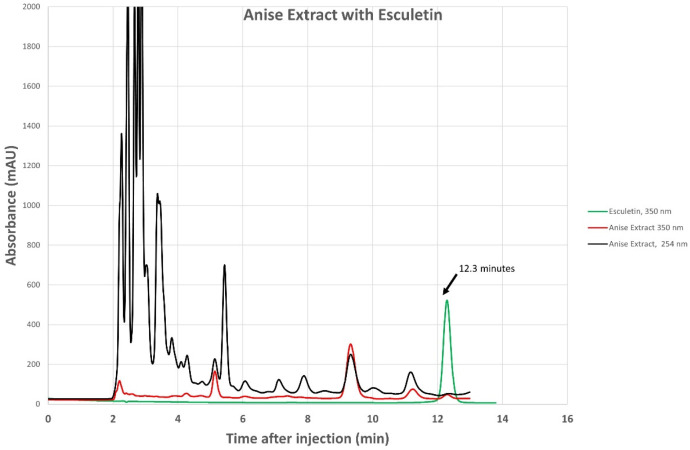
Chromatogram of anise extract monitored at 254 nm (black trace) and at 350 nm (red trace) overlayed using commercial esculetin chromatogram monitored at 350 nm (green trace).

**Figure 2:**
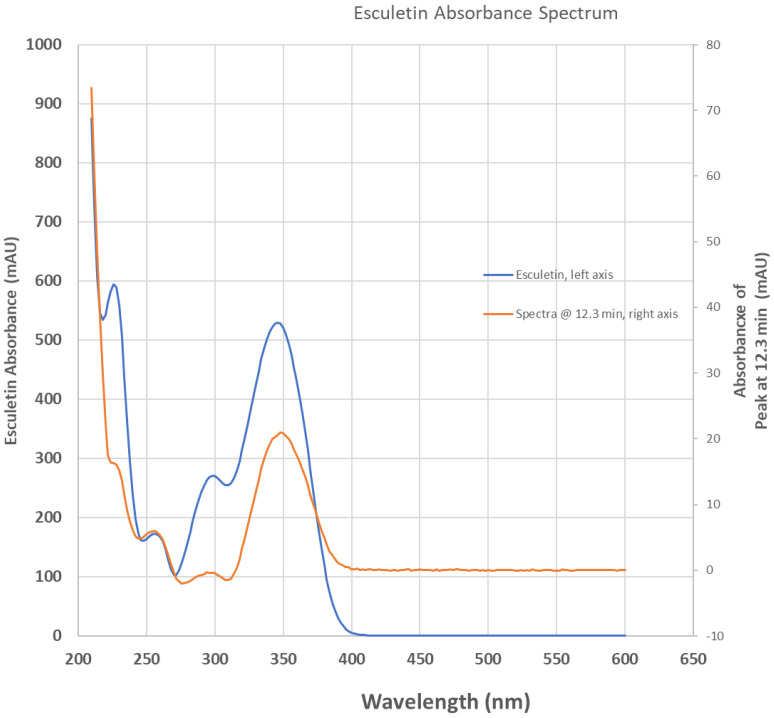
Absorbance spectrum of anise sample extracted at 12.3 min elution time from Figure 1 chromatogram (orange trace, right ordinate axis) overlaying the spectrum of commercial esculetin (blue trace, left ordinate axis).

We confirmed the presence of esculetin in our anise sample using the HPLC technique, as shown in Figure 1. We also reported the chromatogram of anise monitored simultaneously with a photodiode array at 254 nm and fluorescence detector set to 350 nm, overlayed using an esculetin chromatogram monitored at 350 nm, Figure 2. Aromatic compounds are usually detected using UV λ = 254 nm, a wavelength where most chromatographic solvents, including the ones we used, do not absorb UV light. This increases the detected compound’s sensitivity. Comparing the retention time of our sample with the retention times of a known esculetin reference standard, in Figure 1, allowed us to identify esculetin presence based on the similarity of their retention times. Comparing the UV-vis spectrum of our anise sample with that of reference standard esculetin in Figure 2, identifies the presence of esculetin based on the similarity of their UV-vis spectra. Comparison of the UV-vis spectrum and the retention time of known reference sample of esculetin was used to identify the presence of esculetin in the anise sample studied using HPLC.

### 3.2. X-ray Diffraction Analysis

Our studies at low temperature (125 K) agree with earlier crystallographic studies carried out at room temperature for esculetin [36] and 4-methyl-esculetin [37]. Using low temperatures in single-crystal X-ray diffraction analyses allowed us to obtain more accurate geometrical parameters.

#### 3.2.1. Esculetin

**Figure 3:**
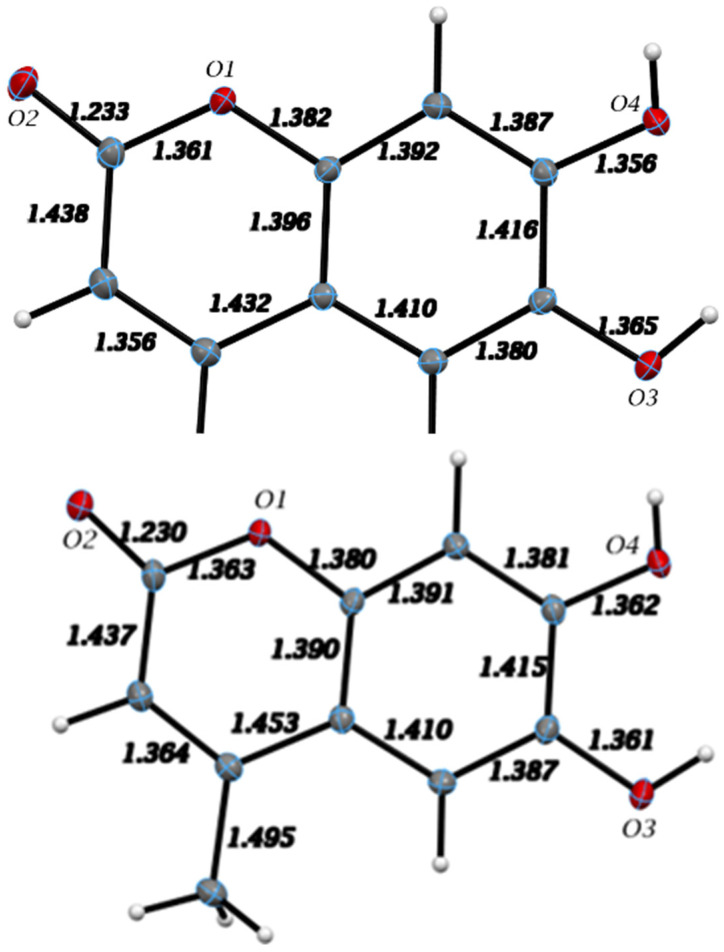
X-ray molecular structure of the esculetin (**top**) and 4-methyl-esculetin (**bottom**) single molecule found in the asymmetric unit of the crystal structure. Atoms are colored according to standard scheme (red = O, grey = C and white = H) Displacement ellipsoids are drawn at the 50% probability level. Distances among heavy atoms are shown.

**Figure 4:**
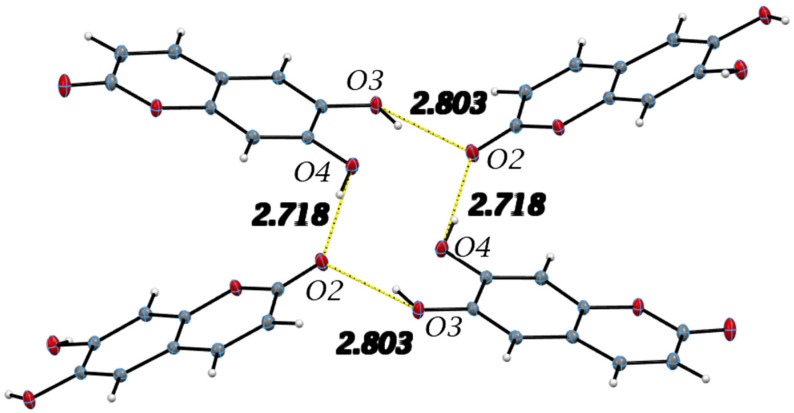
Important intermolecular hydrogen bonds in the esculetin crystal structure and listed in Table 2 are shown. Yellow hydrogen bond distances are used to indicate similar H-bonding pattern to 4-methyl-esculetin in Figure 6. Distances refer to oxygen–oxygen separation.

The single molecule of esculetin in the asymmetric unit of the X-ray structure is shown in Figure 3, top. Distances and angles for the molecule are in agreement with earlier values. The molecule is planar and shown in Figure 4. There are two very strong intermolecular hydrogen bonds, O3-H5⋯O2, 2.8030(9) Å and 154.9(15)° and O4-H6⋯O2, 2.7178(9) Å and 172.4(15)°.

**Figure 5:**
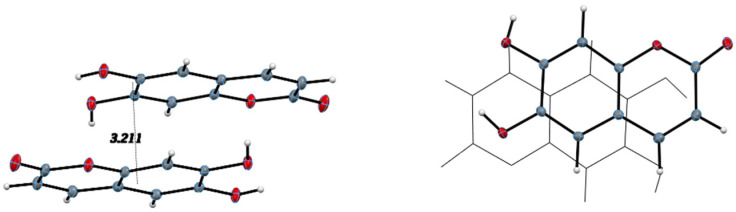
Stacking distance: 3.211 Å between best planes of two inversion related molecules, (**left**); offset stacking down *b*-axis, (**right**).

All hydrogen bond geometrical parameters are listed in Table 2. The coumarin exocyclic oxygen O2 plays a fundamental role in the unit cell packing as the hydrogen bonds involving O2 connect inversion related molecules along the c-axis, as seen in Figure 4. Additionally, the molecules are stacked along the *b*-axis with the stacking distance between best planes of two molecules in the unit cell of 3.211 Å, indicating strong π–π interactions, as seen in Figure 5.

#### 3.2.2. 4-Methyl-Esculetin

**Figure 6:**
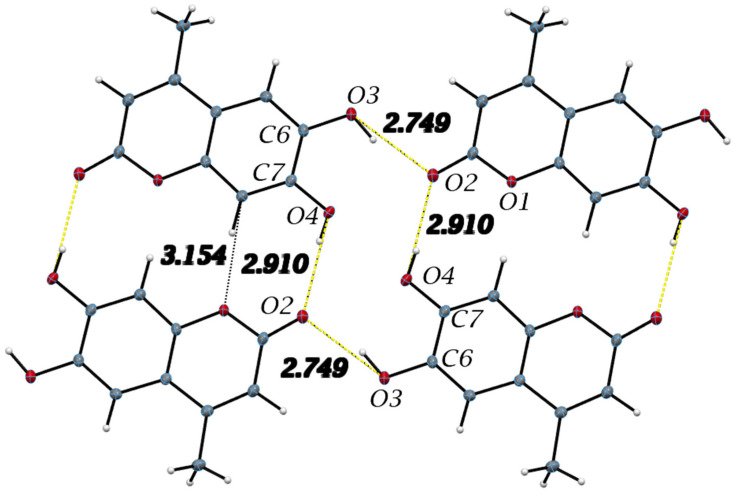
Important intermolecular hydrogen bonding and short contacts listed in Table 3 in 4-methyl-esculetin along the *c*-axis are shown. The distances (Å) are the donor–acceptor values. Hydrogen bonds outlined in yellow are similar those in esculetin in Figure 4.

The single molecule in the asymmetric unit of 4-methyl-esculetin is shown in Figure 3. The geometrical data for the structure are completely consistent with those in Section 3.2.1 for esculetin and, within the estimated standard deviations, present no unexpected values. Interatomic distances are listed in Figure 3, bottom. The molecule, notwithstanding the presence of the methyl group, is planar and shown in Figure 6.

**Table 3:** Hydrogen bond distances (Å) and angles (°) for 4-methyl-esculetin.

	Donor-H	Acceptor-H	Donor–Acceptor	Angle
O3-H6⋯O4	0.90(2)	2.35(2)	2.746(10)	106.8(17)
C8-H8⋯O1 ^#2^	1.043(15)	2.198(15)	3.1544(13)	151.5(11)
O4-H7⋯O2 ^#2^	0.827(17)	2.088(17)	2.9094(11)	172.0(14)
O3-H6⋯O2 ^#1^	0.90(2)	1.87(2)	2.7491(10)	154.9(15)
Symmetry transformations used to generate equivalent atoms:				
#1	*x*, *y*, *z* − 1			
#2	−*x*, −*y* + 2, −*z* + 1			

**Figure 7:**
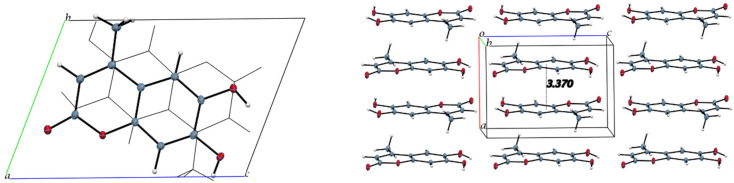
Unit cell packing diagrams. (**Left**), offset stacking of two 4-methyl-esculetin molecules in unit cell, down *a*-axis. (**Right**), view down *b*-axis.

The intermolecular interactions show that molecules of 4-methyl-esculetin are linked by two O–H⋯O and one C–H⋯O hydrogen bonds into a centrosymmetric dimer in a similar hydrogen bonding pattern as esculetin shown in Figure 6 (shown in yellow in Figure 4 and Figure 6). Unlike the esculetin crystal structure, however, the packing of the almost planar 4-methyl-esculetin molecules allows them to be linked by strong π–π interactions into a three-dimensional crystal structure, as seen in Figure 7. The stacking distance between calculated best planes of the ring atoms of the two molecules in the unit cell is 3.370 Å and the efficient offset stacking is seen in the view down the *a*-axis.

In summary, comparison of the packing between esculetin and 4-methyl-esculetin shows that both have similar hydrogen bonding motifs (Figure 4 and Figure 6) involving the exocyclic oxygen atom O2 and the hydroxyl OH atoms, O3H and O4H, of inversion-related molecules. From Figure 5 and Figure 7, we can see that both molecules also both exhibit strong offset molecular stacking between inversion-related molecules, but the methyl derivative shows a more extensive π–π stacking network throughout the crystal as well as the most favorable displaced stacking, such as that seen in graphite.

Stacking interactions are important attractive interactions in chemical and biological systems and of interest to us since both the superoxide anion and the coumarin natural product contain a π system. These interactions are crucial to the maintenance of certain chemical/biological recognition phenomena (including stabilization of protein–substrate contacts and of tertiary structures in biological macromolecules). The factors that govern the geometric outcomes of π–π interactions are not completely understood [38]. We show that these interactions are essential to the antioxidant and scavenging behavior of small natural product molecules having a π system towards the superoxide radical anion. [12,29,31]. Frequently, as in the case of the two coumarins described in our study, hydrogen bonding acts in a cooperative manner to stabilize the intermolecular interactions [39]. Description of the nature of anion–*π* interactions is still much discussed in the literature [40,41,42].

### 3.3. RRDE

The antioxidant capability of anise, esculetin, and 4-methyl-esculetin towards the superoxide radical was studied using RRDE. From earlier studies in our laboratory, we see that the electrochemical RRDE method generation of O_2_^•−^ by O_2_ reduction is an extremely suitable and straightforward method where no byproducts are formed. Overall, it can rapidly detect stable species formed during electrochemical reactions and has high sensitivity and is low cost [24]. We improved the O_2_^•−^ stability by utilizing purified and anhydrous DMSO aprotic solvent. The superoxide radical was generated in a voltaic cell containing anhydrous DMSO as the solvent, tetrabutylammonium bromide, TBAB, as the electrolyte, and by bubbling a controlled amount of oxygen gas, allowing for solution saturation. The superoxide radical was obtained at sufficiently negative potential so that O_2_ could capture an electron from the working disk electrode to form the anionic superoxide radical, O_2_ + e^−^ → O_2_^•−^, Equation (1).

**Figure 8:**
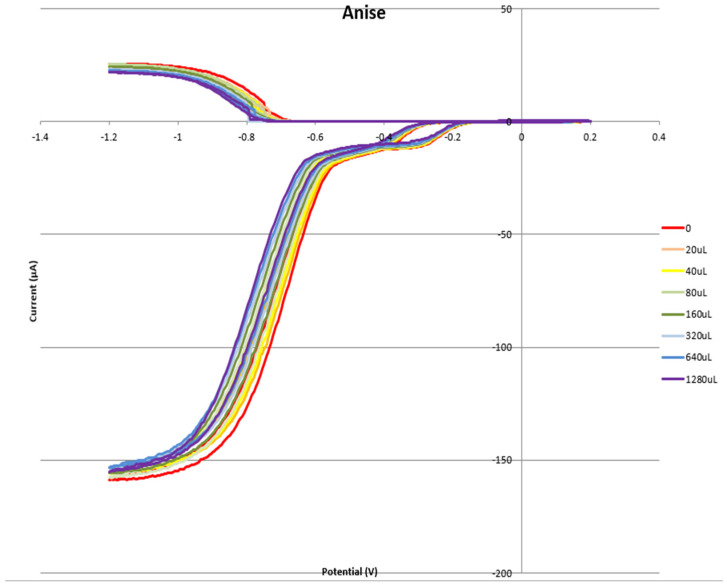
Voltammogram for anise extract.

**Figure 9:**
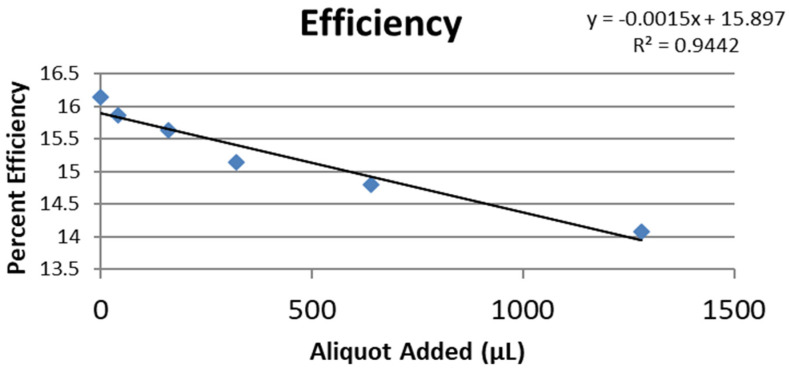
Collection efficiency of anise extract as a function of aliquot added.

**Figure 10:**
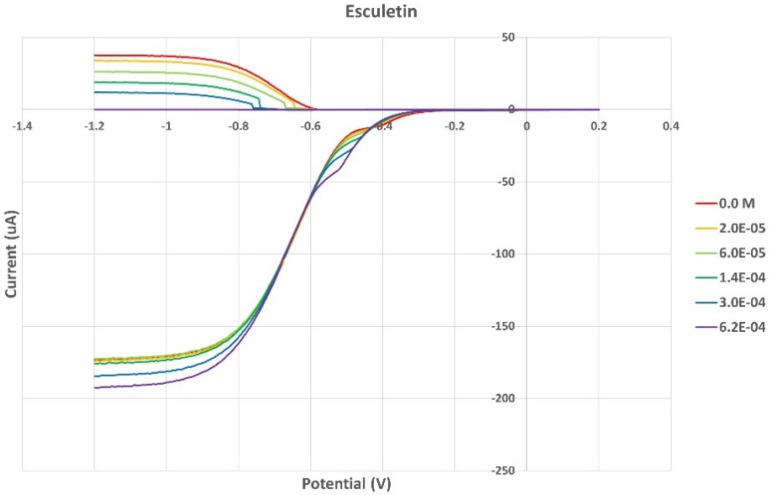
RRDE voltammograms of esculetin at increasing concentrations.

**Figure 11:**
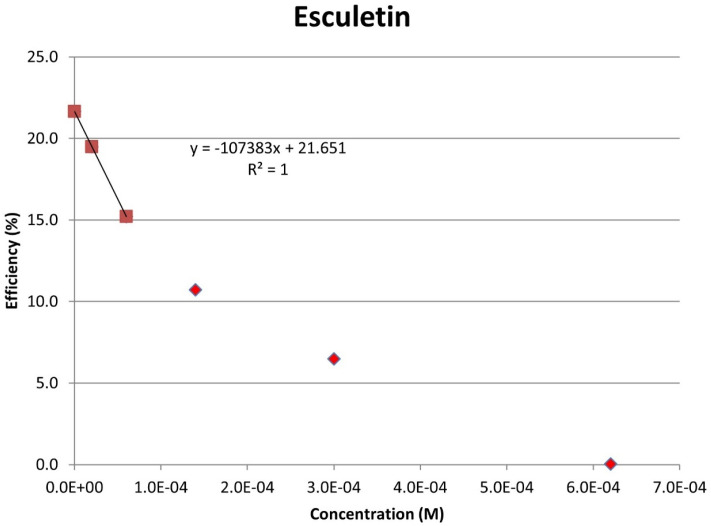
Collection efficiency of esculetin as a function of concentration, linear slope = −10.7 × 10^4^, only for blank plus two initial aliquots (square spots; diamond spots not included in calculation).

**Figure 12:**
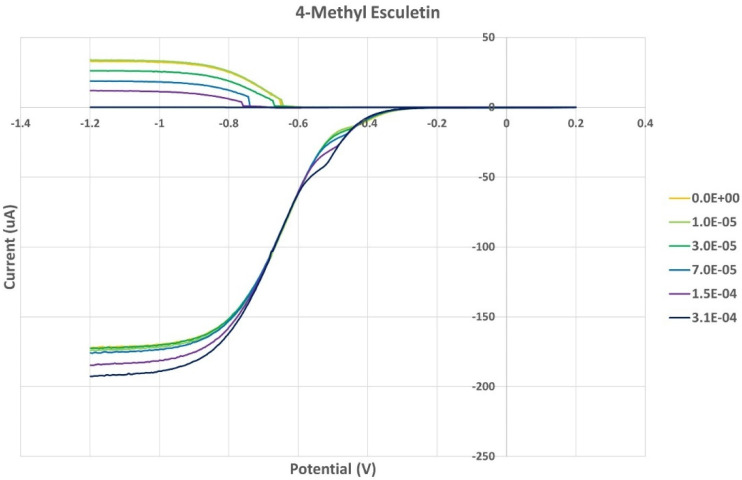
Voltammogram of 4-methyl-esculetin showing total concentration (M).

**Figure 13:**
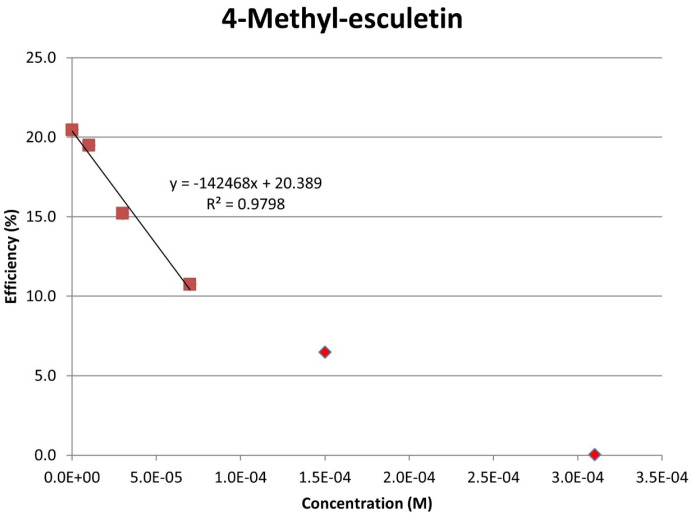
Collection efficiency of 4-methyl-esculetin as a function of concentration, linear slope = −14.2 × 10^4^, considering only blank plus three initial aliquots (square spots; diamond spots not included in calculation).

**Figure 14:**
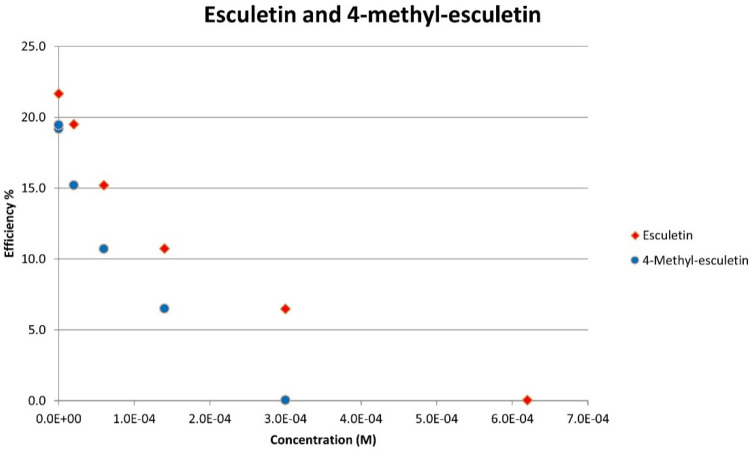
The whole set of aliquots for esculetin (red diamonds) and 4-methyl-esculetin (blue circles), show that both coumarins are able to fully eliminate superoxide radicals around the electrodes. However, 4-methyl-esculetin needs less concentration in the electrovoltaic cell for complete elimination of superoxide (efficiency 0).

Figure 8 shows the RRDE voltammogram of anise, its efficiency is shown in Figure 9, related graphs for esculetin are shown in Figure 10 and Figure 11, and for 4-methyl-esculetin in Figure 12 and Figure 13. A comparison of efficiency plots for the two coumarins is shown in Figure 14.

From the RRDE plot of esculetin and 4-methyl-esculetin, Figure 14, it is seen that as the concentration of the two compounds increases, the superoxide concentration in the voltaic cell decreases to zero. This is specifically located at the upper part of Figure 10 and Figure 12, respectively, and we see that upon adding 620 μL of 0.05 M esculetin (Figure 10) and 0.05 M 4-methyl-esculetin (Figure 12), the superoxide concentration is completely eliminated. In contrast, Figure 8 shows that a large amount of superoxide still exists for the 0.02 g/10 mL anise extract even after adding the 1280 μL aliquot. That is, the signal detected for the anise sample at the ring electrode shows some of the superoxide still remaining and not consumed by the antioxidant. The collection efficiency graph also shows this effect in Figure 9. From comparison of anise slope, studied in this work, to other natural products analyzed using the RRDE method, shown in Table 4, it is seen that anise is a weaker scavenger of the superoxide radical by an order of magnitude. Concentrations of esculetin, 4-methyl-esculetin were 0.05 M, whereas 0.02 g of anise extract was dissolved in 10 mL of DMSO.

It is concluded that the scavenging of anise is weaker than that of olive oil, black seed oil, and propolis but that an anise coumarin component, esculetin, and its 4-methyl derivative, 4-methyl-esculetin, are extremely efficient scavengers.

### 3.4. DFT

**Figure 15:**
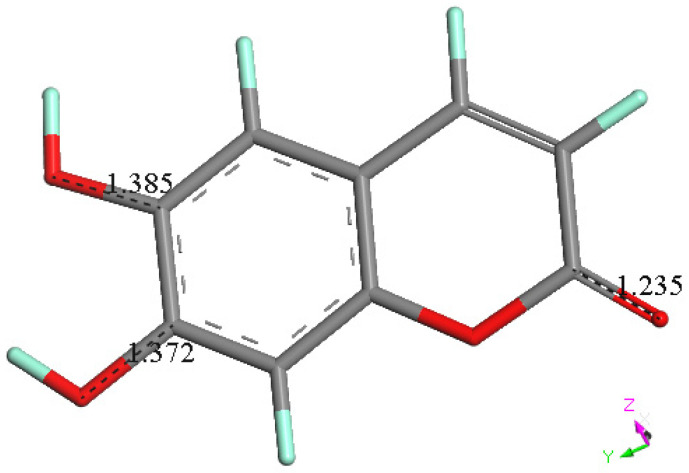
Energy minimum of esculetin, from X-ray atomic coordinates. Selected bond distances are C6-O6, 1.385 Å. C7-O7, 1.372 Å and C2-O2, 1.235 Å.

**Figure 16:**
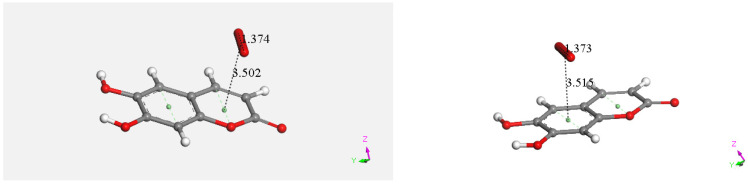
No π–π interactions with superoxide in the pyrone ring, left, nor with the other ring, right, are observed, as upon DFT minimization, the original O-O bond distance of superoxide, 1.373 Å, and the van der Waals separation between superoxide and ring centroids, 3.50 Å, are not modified.

Density functional theory (DFT) technique was applied to consider the reaction mechanism of superoxide scavenging by esculetin. Coordinates of esculetin obtained from the X-ray structure were minimized, and the resulting structure is shown in Figure 15. Since π–π interactions were shown to be important in related studies with flavonoids [11,29], we first decided to explore such an interaction. Therefore, the esculetin pyrone ring centroid of Figure 15 was placed at van der Waals separation, 3.50 Å, from a DFT minimized superoxide radical (O–O bond distance of 1.373 Å) on top of the ring. Upon DFT minimization of the molecular ensemble, the separation between reagent and superoxide anion, and the O–O bond distance of superoxide did not show modification, Figure 16. The O–O bond distance is a crucial marker of superoxide reactivity, since a shorter O–O distance is indicative of molecular oxygen formation. The same behavior was seen for the other ring, Figure 16. It was concluded that both rings of esculetin are not able to establish π–π interactions with superoxide.

**Figure 17:**
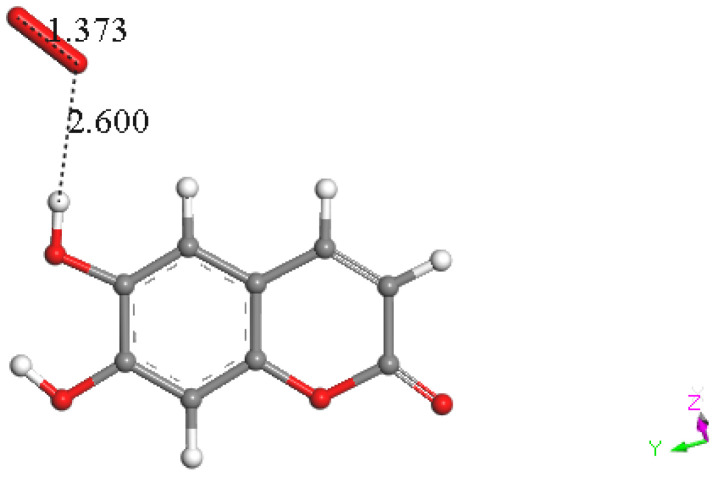
Superoxide approaches H6 (van der Waals separation 2.60 Å) to explore H6 capture by esculetin using DFT.

**Figure 18:**
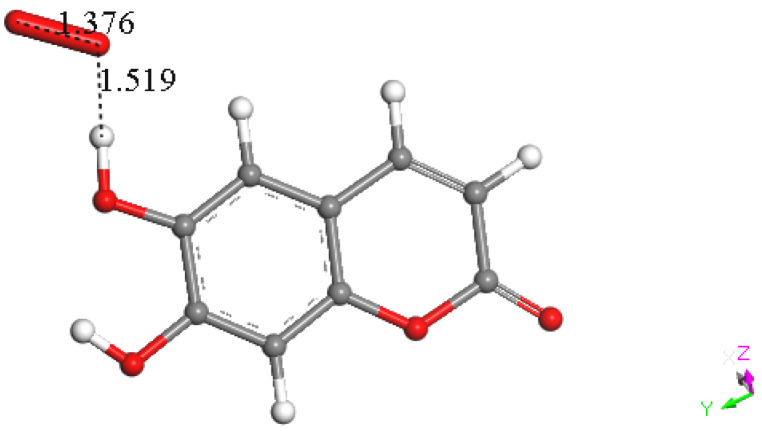
DFT result of DFT minimization of Figure 17 arrangement. No capture of H6 is seen, as O---H (1.519 Å) is longer than the expected bond length, about 1 Å.

Hence, we turned our attention to the other possible mechanism for flavonoids and related polyphenols to react with superoxide through H-atom or proton abstraction; we call this type σ. Therefore, esculetin H6 was placed at van der Waals separation from superoxide, 2.60 Å, Figure 17, and upon DFT minimization, there was no capture of superoxide by H6 either, as seen by O–H distance of 1.519 Å, which is longer than the expected bond length of about 1 Å, Figure 18. However, the H6 proton capture by superoxide may be impeded by an energy barrier, and therefore we explored if the potential expected product having HO_2_ detached from esculetin semiquinone-6 was feasible. Indeed, the DFT minimization of the van der Waals distance separated esculetin semiquinone-6 and HO_2_, 2.60 Å, holds H6 on the HO_2_ species, but it was 0.5 kcal/mol higher energy than that shown in Figure 18. It is concluded that σ proton abstraction is thermodynamically forbidden.

**Figure 19:**
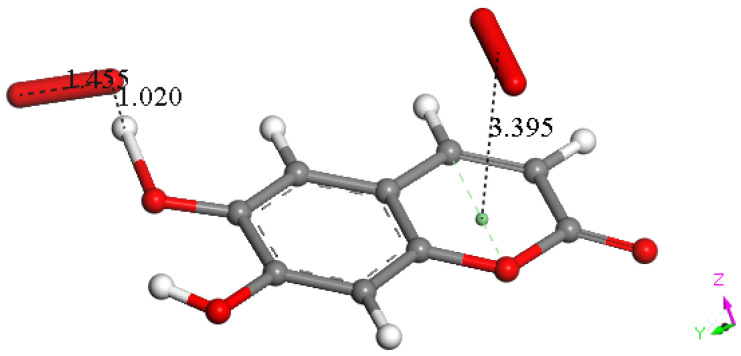
Result of an approached second superoxide (Figure DFT4) upon DFT minimization: H6 is captured (H6-O bond length = 1.020 Å) and the π–π-added superoxide is accepted by the heterocycle ring, 3.395 Å.

**Figure 20:**
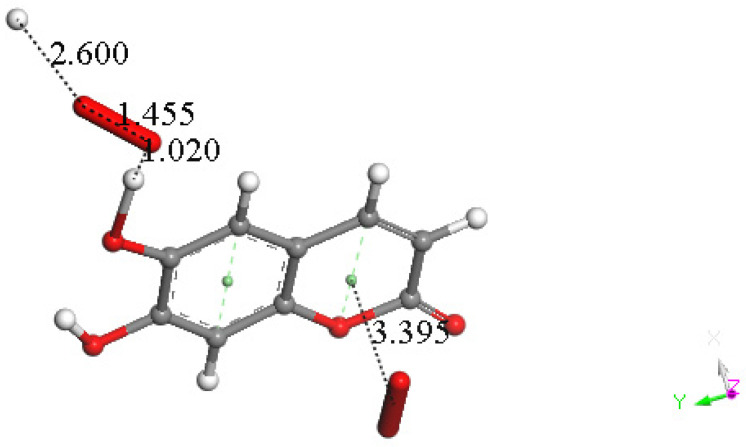
A proton approaches the most exposed oxygen in the HO_2_ moiety of Figure DFT5, with van der Waals separation of 2.60 Å.

**Figure 21:**
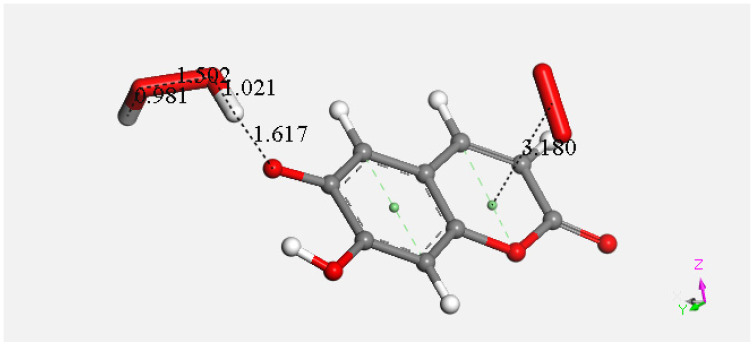
After DFT minimization of Figure 20 arrangement H_2_O_2_ forms.

**Figure 22:**
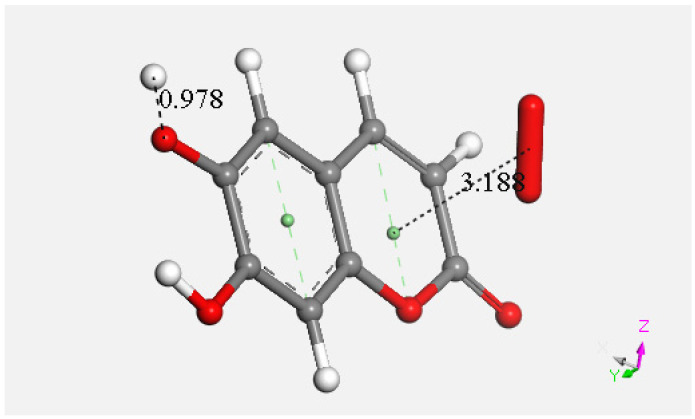
Last DFT shows formation of η-O_2_-esculetin. This results from the sequence (1), after H_2_O_2_ elimination from Figure DFT5; (2) proton approaching O6 (2.60 Å, O6-H6 formation (0.978 Å). The final product is the esculetin-η-O_2_ species.

At this point, we were very interested in esculetin, as this scavenger seemed unusual. Indeed, other scavengers analyzed by us in previous studies showed that both ways (σ and π) could be possible as initial steps of scavenging, in contrast with esculetin, which seems to be more limited in its options for scavenging. Therefore, we decided (to be more precise, there were no other options) to further explore the only product that seemed possible for scavenging, the arrangement shown in Figure 18. Thus, the attack of a π–π-approached superoxide, Figure S1, was attempted, and this was successful after DFT minimization, Figure 19, which shows the π–π superoxide incorporated by the pyrone ring, 3.395 Å, and H6 captured by superoxide σ, H6-O bond length 1.020 Å. It is clear that the arrangement shown in Figure 19 must be an intermediate, as it is a nonradical 2-anion, which is the result of interaction between esculetin and two superoxide radicals. It seemed logical to interact this species with a proton, at least. Such a reaction was performed by approaching the proton at van der Waals separation of 2.60 Å to the most exposed O of the first superoxide reacted, Figure 20, and then performing DFT minimization on the assemblage. This result is presented in Figure 21, which shows H_2_O_2_ formation, separated at 1.617 Å distance from the aromatic moiety. Figure 21 also shows the π–π-reacted superoxide still engaged with the pyrone ring with separation between centroids of 3.180 Å. The next step in our investigation consisted in elimination of H_2_O_2_ and so the remaining aromatic moiety (a −1 anion) was approached by a second proton towards O6, Figure S2, and after DFT minimization, a final neutral product, Figure 22, shows formation of η-O_2_-esculetin. That is, the π–π-added superoxide is still engaged in bonding with 3.183 Å separation between the centroids of the superoxide and esculetin, shorter than the van der Waals value of 3.50 Å.

As seen in Figure S3, 4-methyl-esculetin works the same way regarding superoxide scavenging when compared with Figure 19.

In conclusion, the behavior of scavengers mimicking SOD [11] is described by Equation (3) and is in contrast to that of esculetin. Esculetin sequesters the second superoxide, which remains π–π-trapped by the heterocyclic ring and generates H_2_O_2_ after proton capture to form esculetin-η-O_2_ as a reaction product (Equation (4)).

2 O_2_^•−^ + 2 H^+^ → H_2_O_2_ + O_2_ (3)

2 O_2_^•−^ + 2 H^+^ + esculetin → H_2_O_2_ + esculetin-η-O_2_ (4)

The RRDE study indicates that anise shows weak antioxidant activity. Pure compounds esculetin and 4-methyl-esculetin showed much higher antioxidant activity than the anise sample, both being able to decrease the efficiency to zero for 140 μM aliquot at a concentration of 6.0 × 10^−4^ M and 3.0 × 10^−4^ M, respectively; that is, complete elimination of superoxide was obtained using the pure compounds. The slopes of the collection efficiency graphs indicated that 4-methyl-esculetin is a stronger scavenger than esculetin, slopes of −14.2 × 10^4^ and −10.7 × 10^4^, respectively, see Figure 11 and Figure 13. DFT studies showed that one molecule of esculetin is able to scavenge two superoxide molecules by stabilizing one superoxide radical on top of its heterocyclic ring and converting the other into H_2_O_2_.

It is known that the superoxide anion can undertake a surprising number of chemical reactions [24]. Besides the disproportionation reaction that is evident through SOD intervention, the superoxide anion can also assume one-electron transfer, deprotonation, and nucleophilic substitution reactions. Because many organic molecules can behave as a proton source, the superoxide ion O_2_^•−^ can react with a proton, H^+^, or a proton donor, HX, to form HO_2_^•^ through proton abstraction. Additionally, an important review [36] described the different geometries observed, both experimentally and computationally predicted, demonstrating anion–π interactions. That anions can be found directly “on-top” of planar aromatic ring structures is known. The arrangement whereby the superoxide anion resides on top of the centroid of the esculetin (and 4-methyl-esculetin) is supported by our computational DFT results. The final product is the neutral, nonradical π complex, the esculetin-η-O_2_ structure.

## 4. Conclusions

As determined using the RRDE method, the scavenging of superoxide by anise is one order of magnitude weaker than for olive oil [35], black seed oil [33], and propolis [30]. In contrast, the slope of a coumarin component of anise, esculetin (−10.2 × 10^4^), shows it to be an excellent antioxidant, slightly weaker than butein (−11.2 × 10^4^) [31]. Esculetin showed unprecedented complete elimination of superoxide in the voltaic cell, as shown in Figure 13. Moreover, 4-methyl-esculetin exhibited even stronger antioxidant scavenging of superoxide than that of esculetin with RRDE collection efficiency slope of −14.2 × 10^4^ Figure 14. The exceptional radical scavenging behavior (against DPPH and galvinoxyl radicals) by 4-methyl-esculetin was previously recognized by Pedersen, et al. [14] in a study of twenty-two 4-methylcoumarin compounds. In the same work, 4-methyl-esculetin also showed the largest decrease in ROS production in L6 myoblasts, even at very low concentrations.

Comparison with scavenging behavior by other coumarins as calculated using DFT methods shows that subtle structural differences in the coumarin framework may imply marked differences in scavenging. For instance, when the catechol moiety of esculetin (position 6,7) is shifted to position 7,8 in 4-methyl-7,8-dihydroxy coumarin, that coumarin shows a superoxide dismutase action, which, beside H_2_O_2_ formation, includes the formation and elimination of a molecule of O_2_ [12]. In this work, we found that the mode of esculetin scavenging of superoxide radicals is similar to the behavior of butein, which formed a π–π butein-η-O_2_ product [31]. Esculetin sequestered superoxide and generated H_2_O_2_ after proton capture. In addition, a second superoxide was π–π-trapped by the heterocycle ring and a π–π esculetin-η-O_2_ reaction product was formed.

From the X-ray diffraction studies of esculetin and 4-methyl-esculetin, we saw that the intermolecular interactions for the two compounds are very similar, even if they crystallize in different space groups. They both have a very strong intermolecular hydrogen bonding arrangement, and both exhibit strong offset molecular stacking between inversion-related molecules, as seen from the shorter-than-van der Waals distances between planes of both molecules in the crystal structure. The 4-methyl-esculetin showed a more extensive π–π stacking network throughout the crystal and a displaced stacking motif such as that seen in graphite. This subtle difference in crystal packing motif might help explain the experimentally observed better scavenging action of 4-methyl-esculetin towards the superoxide anion.

The interesting and varied chemical nature of coumarin derivatives and how these derivatives correlate with pharmacological and medicinal properties has been reported in several foundational reviews [16,43,44,45,46,47]. Current reviews that describe coumarin scavenging of superoxide include references [48,49]. In addition, the antioxidant abilities of coumarin metal complexes to scavenge free radicals recently has been reported in an excellent review [50]. Furthermore, several reports of coumarin-based bioinorganic compounds include phenylselenyl derivatives that act as sensors for selective detection of superoxide [51] as well as ruthenium coumarin complexes which scavenge superoxide and DPPH radicals [52] and other coumarin metal complexes made with ruthenium, rhodium and iridium showing antioxidant activity [53]. 

In this work, the combination of experimental electrochemistry, X-ray diffraction, and quantum chemistry was shown to be an effective strategy to rationalize the association between structure and antioxidant activity. Because the coumarin framework is of importance to many areas, we will continue our investigations into the relationship between bio/chemical antioxidant activity when relatively small changes are made to the coumarin molecular structure.

## Figures and Tables

**Table 1:** Crystal data.

Crystal Data	Esculetin	4-Methyl-esculetin
Chemical formula	C_9_H_6_O_4_	C_10_H_8_O_4_
*M*_r_, g/mol	178.14	192.16
Crystal system, space group	Monoclinic, *P*2_1_/*c*	Triclinic, *P* − 1
Cell parameters, *a*, *b*, *c* (Å)	8.2707 (5), 6.7867 (4), 13.2033 (7)	6.7550 (6), 7.1429 (6), 9.5517 (8)
α (°)	90	68.166 (1)
β (°)	103.558 (1)	85.275 (1)
γ (°)	90	69.616 (1)
*V* (Å^3^)	720.46 (7)	400.43 (6)
*Z*	4	2
Density (calculated)	1.642 g/cm^3^	1.594 g/cm^3^
Absorption coefficient, μ (mm^−1^)	0.132	0.125
F(000)	368	200
Crystal size (mm)	0.150 × 0.190 × 0.340	0.100 × 0.170 × 0.270
*T*_min_, *T*_max_	0.981, 0.957	0.988, 0.967
No. of measured, independent and observed [*I* > 2σ(*I*)] reflections	17,336, 2201, 2027	9707, 2339, 1861
*R* _int_	0.0238	0.0205
*R*[*F*^2^ > 2σ(*F*^2^)], *wR*(*F*^2^), *S*	0.036, 0.109, 1.032	0.0397, 0.1107,1.110
No. of reflections	2201	2339
No. of parameters	142	160
Δρ_max_, Δρ_min_ (e Å^−3^)	0.538, −0.277	0.472, −0.280

**Table 2:** Hydrogen bond distances (Å) and angles (°) for esculetin.

	Donor-H	Acceptor-H	Donor–Acceptor	Angle
C7-H7⋯O3 ^#2^	0.995(14)	2.573(14)	3.5362(10)	162.8(11)
O4-H6⋯O2 ^#1^	0.845(16)	1.877(16)	2.7178(9)	172.4(15)
O3-H5⋯O4	0.872(18)	2.216(16)	2.6946(9)	114.3(13)
O3-H5⋯O2 ^#4^	0.872(18)	1.989(18)	2.8030(9)	154.9(15)
C4-H4⋯O1 ^#3^	1.003(13)	2.508(13)	3.4952(10)	167.8(11)
Symmetry transformations used to generate equivalent atoms:				
#1	−*x* + 1, *y* + 1/2, −*z* + 1/2			
#2	*x*, −*y* + 3/2, *z* − 1/2			
#3	*x*, −*y* + 3/2, *z* + 1/2			
#4	*X* + 1, −*y* + 3/2, *z* + 1/2			

**Table 4:** Comparison of slopes of anise, studied in this work, and other natural products analyzed using the RRDE method.

Olive Oil [35]	Black Seed Oil [33]	Propolis [30]	Anise (This Work)
−0.0838	−0.078	−0.0864	−0.0015

## Data Availability

Crystal data of esculetin and 4-methylesculetin have been deposited at the Cambridge Structural Database (CSD) and are available at https://www.ccdc.cam.ac.uk/structures, 2322199, 2322200 (accessed on 23 December 2023).

## Supplementary Materials

The following supporting information can be downloaded at: https://www.mdpi.com/article/10.3390/ph17010067/s1, Figure S1. A 2nd superoxide is approached to the pyrone ring of Figure 18 arrangement; Figure S2. After elimination of H_2_O_2_ from Figure 21 arrangement a proton is approached to O(polyphenol), O7; Figure S3. 4-Methyl-esculetin works the same way regarding superoxide scavenging, see Figure 19.
