# Supplementary material for: Examining the Antioxidant and Superoxide Radical Scavenging Activity of Anise, (Pimpinella anisum L. Seeds), Esculetin, and 4-Methyl-Esculetin Using X-ray Diffraction, Hydrodynamic Voltammetry and DFT Methods
Source: Pharmaceuticals (Basel). 2023 Dec 31;17(1):67. doi: 10.3390/ph17010067 (PMC10818671; doi:10.3390/ph17010067)
Supplement: Supplementary file 1 [file pharmaceuticals-17-00067-s001.zip › pharmaceuticals-2779364-supplementary.pdf]

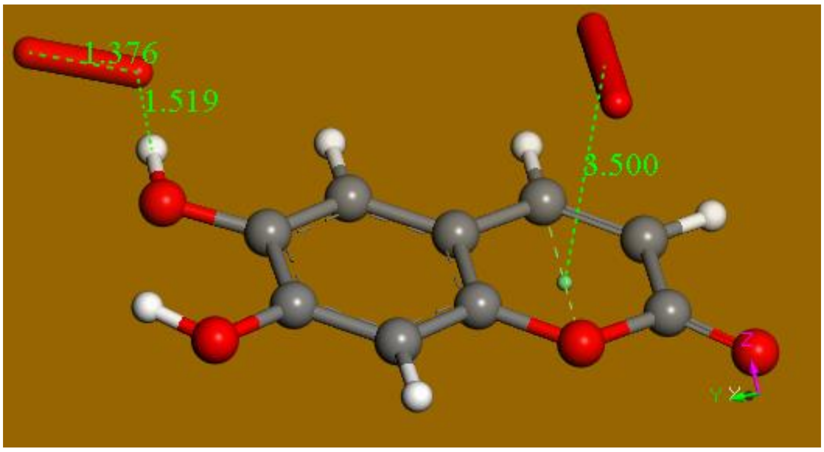

Figure S1. A 2nd superoxide is approached to the pyrone ring of Figure 18 arrangement.

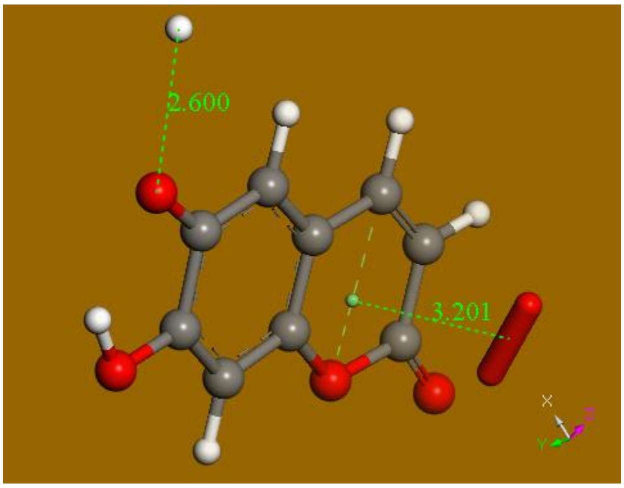

Figure S2. After elimination of H_2O_2 from Figure 21 arrangement a proton is approached to O(polyphenol), O7.

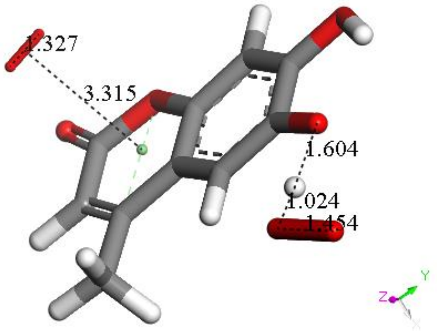

Figure S3. 4-Methyl-esculetin works the same way regarding superoxide scavenging, see Figure 19.
